# Peritoneal Metastases From Colorectal Cancer: Defining and Addressing the Challenges

**DOI:** 10.3389/fonc.2021.650098

**Published:** 2021-03-16

**Authors:** Onno Kranenburg, Kurt van der Speeten, Ignace de Hingh

**Affiliations:** ^1^Department of Surgical Oncology, University Medical Center Utrecht, Utrecht, Netherlands; ^2^Utrecht platform for Organoid Technology, Utrecht University, Utrecht, Netherlands; ^3^Department of Surgical Oncology, Faculty of Medicine, Biomedical Research Institute (BIOMED) Research Institute, Hospital Oost-Limburg, Belgium and University Hasselt, Diepenbeek, Belgium; ^4^Department of Surgery, Research School for Oncology and Developmental Biology, Catharina Hospital Eindhoven, The Netherlands and Maastricht University, Maastricht, Netherlands

**Keywords:** colorectal, CMS4, peritoneal, imaging, organoid

## Abstract

The presence of peritoneal metastases (PM) in patients with colorectal cancer (CRC) is associated with an extremely poor prognosis. The diagnosis of PM is challenging, resulting in an underestimation of their true incidence. While surgery can be curative in a small percentage of patients, effective treatment for non-operable PM is lacking, and clinical and pre-clinical studies are relatively sparse. Here we have defined the major clinical challenges in the areas of risk assessment, detection, and treatment. Recent developments in the field include the application of organoid technology, which has generated highly relevant pre-clinical PM models, the application of diffusion-weighted MRI, which has greatly improved PM detection, and the design of small clinical proof-of-concept studies, which allows the efficient testing of new treatment strategies. Together, these developments set the stage for starting to address the clinical challenges. To help structure these efforts, a translational research framework is presented, in which clinical trial design is based on the insight gained from direct tissue analyses and pre-clinical (organoid) models derived from CRC patients with PM. This feed-forward approach, in which a thorough understanding of the disease drives innovation in its clinical management, has the potential to improve outcome in the years to come.

## Introduction

Colorectal cancer (CRC) is one of the most common forms of cancer and the second leading cause of cancer-related mortality in the Western world. Death from CRC is virtually always the consequence of metastatic spread to distant sites in the body such as the liver, the peritoneal cavity, and the lungs. Patients with metastases in the peritoneal cavity (peritoneal metastases, PM) have the worst prognosis (1, 2).

In general, PM are under-diagnosed as their detection with routine imaging protocols is difficult, due to their small size and limited contrast resolution in soft tissues (3, 4). The true incidence of PM is therefore unclear, although in autopsy series it was reported to be as high as 40–80% (5, 6) (Table 1). The development of PM in CRC patients is often associated with a rapidly declining performance status, involving recurrent bowel obstruction, the formation of malignant ascites, visceral pain, and malnutrition (Table 1) (7). In most cases this precludes surgery and systemic therapy, leaving only palliative care to ensure the best possible quality of life. When left untreated, the median overall survival of this patient group is ~5 months (8). The benefit of systemic chemotherapy is dramatically reduced in the subgroup of CRC patients with PM (2, 9), and their poor visualization complicates assessment of their response to treatment. In the past decade, this has resulted in the active exclusion of patients with PM from clinical trials (10). Taken together, CRC with PM is a very common and highly aggressive, but under-diagnosed and under-studied disease entity.

**Table 1:** Incidence of PM and disease burden.

**Incidence**	
Synchronous	5–8%^*^
Metachronous	4–19%^*^
At autopsy	up to 80%
**Burden of disease**	
Bowel obstruction	Biliary or uretral obstruction
Abdominal pain	Anorexia
Nausea	Cachexia
Ascites	Fatigue
Enteric fistulas	

* *Due to the poor performance of routine imaging procedures these values are likely underestimations*.

**Figure 1:**
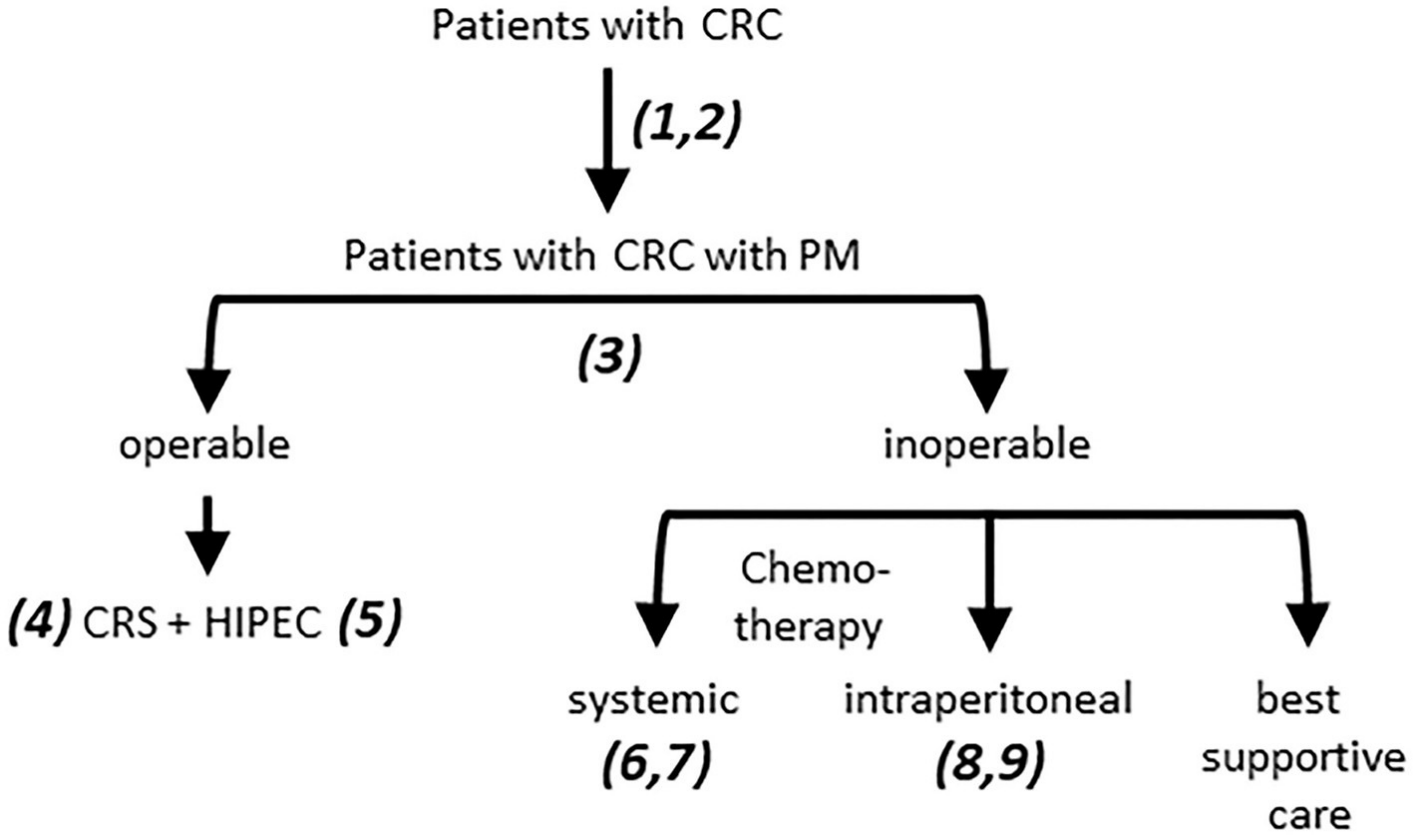
Challenges in the treatment of PM from CRC. 1. Patients with PM are underdiagnosed due to poor performance of routine imaging procedures. 2. Risk assessment of metachronous PM development is insufficient. This requires a comprehensive analysis of clinical parameters and (epi)genetic features of tumors and patients, informing the development of (composite) biomarkers. 3. 40–60% of the patients currently selected for CRS HIPEC experience rapid disease recurrence and are over-treated. The rate of under-treatment (patients who would have benefitted but were not selected) is unknown. Improved patient selection requires better imaging modalities for robust PCI assessment, combined with clinical and (epi-)genetic features, as in 2. 4. Incomplete resection leaves tumor residue which may initiate intra-abdominal recurrence. Novel intra-operative imaging strategies are needed to guide CRS. 5. The currently used monotherapies in HIPEC are unlikely to be effective. Novel more effective treatment strategies need to be developed. 6. Patients with PM benefit least from modern chemotherapy regimens. The factors determining PM resistance to systemic therapy need to be identified. 7. Some patients with PM do benefit from long-term chemotherapy. Biomarkers predicting such benefit are urgently needed. 8. The currently used monotherapies in i.p. chemotherapy are unlikely to be effective. Novel more effective treatment strategies need to be developed. 9. Response assessment to i.p. chemotherapy is challenging due to underperformance of CT. Improvement requires development of better imaging modalities. CRC, colorectal cancer; PM, Peritoneal metastases; CRS, cytoreductive surgery; HIPEC, hyperthermic (heated) intraperitoneal chemotherapy.

**Figure 2:**
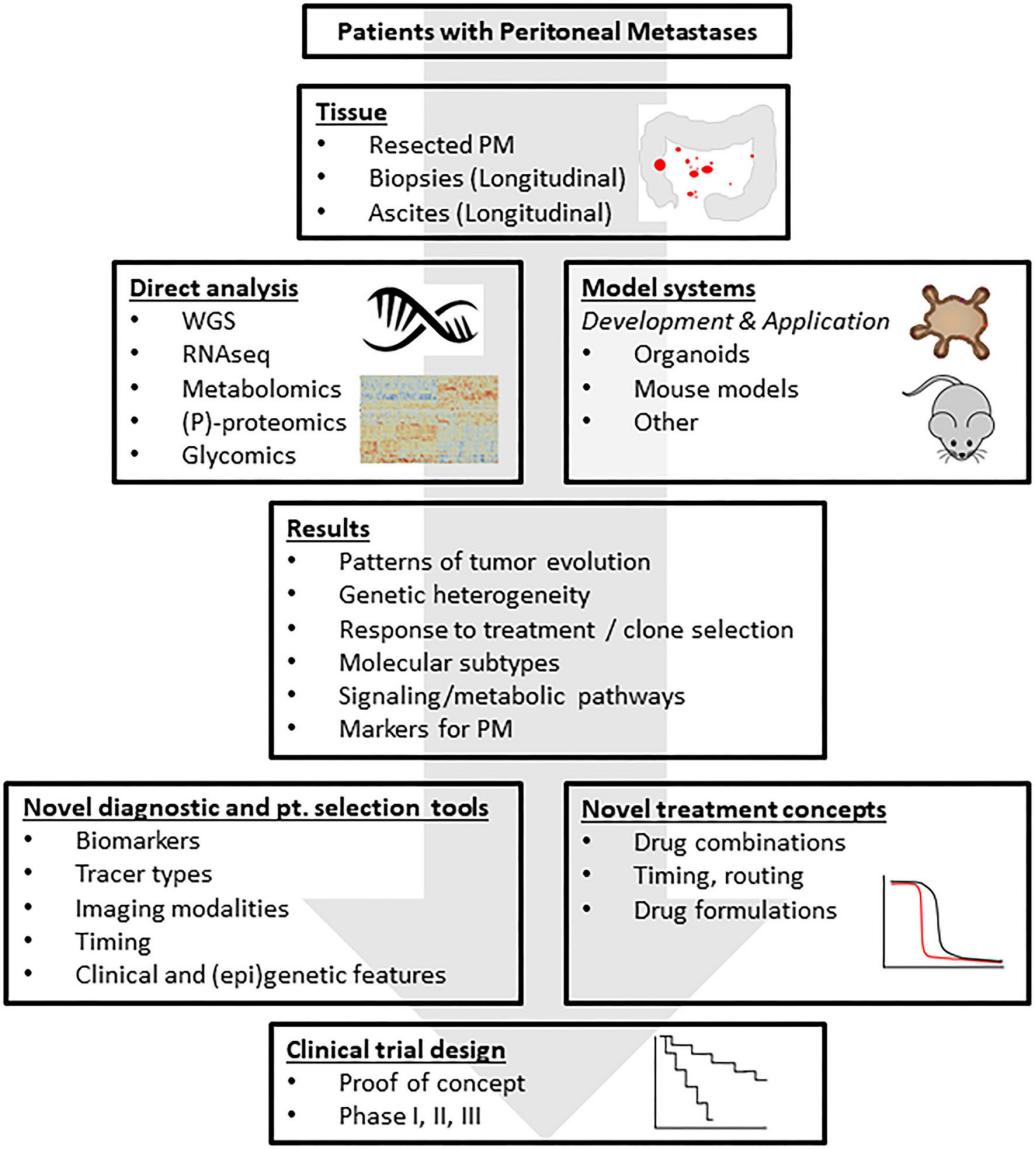
A research framework addressing the challenges. The standardized collection of tissue and body fluids derived from patients with peritoneal metastases from CRC generates biobanks of frozen and fixed tissues for downstream molecular analysis. Moreover, organoid technology allows the co-establishment of “living biobanks” in which individual cancer patients are represented by their tumor-derived organoids. These organoids may be used in transplantation studies generating spontaneous metastasis models in mice. The molecular tissue analyses will generate novel leads for the detection and treatment of PM, for understanding resistance mechanisms, tumor cell plasticity and intra- and inter-lesion (epi-)genetic heterogeneity. Insight into the biology of PM will lead to the formulation of novel treatment concepts that can subsequently be tested in the generated novel PM model systems. These efforts should yield a series of pre-clinically validated novel treatment strategies, possibly limited to specific, identifiable patient subgroups. These strategies can then be tested in small proof-of-concept studies to generate biological proof for the validity of the treatment concept in cancer patients, and subsequently in regular phase 1–3 clinical trials. Analysis of the tissues from such trials may subsequently identify potential resistance mechanisms and help design pre-clinical studies that are aimed at further improving the strategy. This translational feed-forward approach has the potential to impact clinical outcome in the years to come. PM, peritoneal metastases; WGS, whole genome sequencing; RNAseq, RNA sequencing; (P)-proteomics, (phospho-)proteomics.

In this report we provide an overview of the major challenges in the field of PM from CRC (Figure 1) and describe a research framework that addresses these challenges (Figure 2). The central premise is that innovative and more effective treatment concepts can only be designed on the basis of a thorough understanding of the disease.

## Diagnosis

Several approaches are used to diagnose and quantify PM from CRC (Table 2). The peritoneal cancer index (PCI; a semi-quantitative measure of intraperitoneal tumor load) is the golden standard for assessing the extent of PM, and an important tool for selecting patients for potentially curative cytoreductive surgery (CRS; see below). Exploratory laparoscopy is performed prior to CRS in case extensive PM is suspected, but this procedure leaves important intra- and extra-peritoneal regions unexamined (11). Pre-operative non-invasive imaging methods for determination of the PCI in patients with (suspected) PM are therefore urgently needed (Figure 1). Typically, the size of the individual peritoneal tumor nodules is below the detection level of conventional CT or PET. Indeed, with the current selection strategies CRS is discontinued in up to 40% of the procedures due to a high PCI score (12, 13). More recently it was shown that diffusion-weighted MR imaging (DW-MRI) outperforms other imaging techniques with regards to sensitivity and specificity (14–16). DW-MRI also detects extra-peritoneal disease such as occult liver metastases with high sensitivity. Finally, a recent study employed molecular imaging using Fibroblast Activation Protein (FAP) inhibitor (FAPI) as a PET tracer for the detection of PM and demonstrated superiority over standard FDG-PET (17). The basis for FAP-based detection of PM is presumably related to the generally high amount of FAP-positive cancer-associated fibroblasts (CAFs) in these lesions.

**Table 2:** Diagnostic tools.

Diagnostic laparoscopy	Current golden standard to determine preoperative PCI and obtain pathological proof. However, may result in iatrogenic bowel lesion due to adhesions resulting from previous surgery or tumor nodules. These adhesions may also limit visualization of the peritoneum and hamper appropriate determination of the PCI.
CT	Poor performance due to small lesion size and limited contrast resolution.
FDG-PET	Poor performance due to small lesion size, limited contrast resolution especially in mucinous tumors.
FAPI-PET	Experimental. Superior over FDG-PET. Based on the detection of reactive stroma rather than the tumor cells themselves.
Diffusion-weighted MRI	Highest sensitivity to detect PM of all currently available imaging modalities. May outperform DLS with regard to safety and completeness of visualization.

*PCI, Peritoneal Cancer Index; CT, computed tomography; FDG-PET, fluorodeoxyglucose-positron emission tomography (PET); MRI, magnetic resonance imaging*.

Prospective studies should now be designed to assess whether DW-MRI or FAPI-PET are indeed superior modalities for the diagnosis and quantification of PM and for selecting patients for CRS.

## Risk Assessment

PM can be detected at first diagnosis of CRC (synchronous PM), but they can also develop in the months or years after primary tumor resection (recurrent/metachronous PM) (18, 19). Risk factors for developing *recurrent* PM are the presence of synchronous PM, age (<60 years), a T4 primary tumor, location of the primary tumor in the proximal colon, the presence of activating mutations in *BRAF*, a mucinous or signet ring cell histology, and a CMS4 molecular subtype (1, 5, 20–24). Some of these features have been used to select patients for second-look surgery (22, 23, 25–28), but validated approaches to select patients at risk for an alternative more aggressive treatment strategy are currently not available.

Novel biomarkers predicting PM development with high accuracy are urgently needed (Figure 1). These could be based on the genetic or biological trait(s) of the resected primary tumor. In addition, genetic and non-genetic variation in the patient population is also likely to determine the risk of developing PM, independently of tumor-intrinsic variables (29). Alternatively, the ultra-sensitive detection of tumor-derived biological material (e.g., cell-free tumor DNA, RNA, or protein) that is shed by microscopic tumor deposits could also serve as biomarkers predicting recurrence. For instance, the detection of circulating tumor DNA (ctDNA) in plasma identifies stage II and stage III patients at high risk of developing (any) distant metastases (30–33). However, plasma ctDNA levels are extremely low in patients with PM. Rather, PM-derived ctDNA can readily be measured in peritoneal fluid, offering an alternative source of biomarkers reporting on the potential presence of micrometastases in the peritoneum (34). Indeed, the presence of cancer-specific molecular biomarkers in peritoneal fluid predicts PM formation in gastric and pancreas cancer (35, 36).

## Treatment

An overview of the currently available treatment modalities for operable and inoperable PM is shown in Table 3.

**Table 3:** Treatment modalities.

**Operable**	**Remarks**
Cytoreductive surgery (CRS) plus hyperthermic intra-peritoneal chemotherapy (HIPEC)	Only in a selected subgroup of patients with limited intraperitoneal disease burden. Added value of HIPEC remains unproven in patients with PM from CRC. Rationale for drug choice is lacking.
Systemic chemotherapy and targeted therapy	PM appear to be relatively refractory to systemic therapy.
Immune checkpoint inhbitors	Only in patients with mismatch repair-deficient (dMMR) tumors
**Inoperable**	**Remarks**
Pressurized intra-peritoneal aerosolized chemotherapy	Experimental. Prospective studies showing benefit of this procedure are lacking.
Repeated intra-peritoneal infusion of chemotherapy	Experimental. Prospective studies showing benefit of this procedure are lacking.

### Adjuvant Treatment

The development of biomarkers predicting PM formation after primary tumor resection calls for the design of effective adjuvant treatment strategies. Currently, the options for adjuvant therapy are limited. Adjuvant systemic chemotherapy for patients with high-risk stage II and stage III tumors reduces the risk of recurrence by only ~15%. Patients with PM are even less likely to benefit from systemic treatment, possibly due to low penetration of systemically delivered chemotherapy in the peritoneal cavity (2, 9). Therefore, the application of adjuvant intra-peritoneal chemotherapy following primary tumor resection is an attractive alternative local approach, aiming to kill any microscopic disease inside the peritoneum (27, 28). Although this adjuvant strategy recently failed to show benefit using heated oxaliplatin for 30 min (27, 28) it should be re-considered once effective intra-peritoneal chemotherapeutic regimens are identified. Such regimens are currently lacking (Figure 1). Novel PM culture systems based on organoid technology have recently become available and are now being used to design and test novel effective treatments (37, 38). Importantly, organoid technology represents the only available platform for predicting therapy response in individual patients with GI cancers (39–43).

### Cytoreductive Surgery and Hyperthermic Intraperitoneal Chemotherapy

Once PM are diagnosed, the only treatment that results in long-term survival involves radical surgery to remove all visible disease (CRS). The addition of heated intra-peritoneal chemotherapy (HIPEC), may further improve survival (44, 45). This procedure was first introduced by Paul Sugarbaker for the treatment of Pseudomyxoma Peritonei (PMP) (46) and has since been widely adapted to treat PM from multiple cancer types. However, the added value of HIPEC over CRS alone for the treatment of colorectal PM has recently been questioned in the PRODIGE7 trial (47). CRS with or without HIPEC is a high-cost procedure that is associated with considerable morbidity and mortality rates (48, 49). Therefore, careful patient selection is essential. Prognostic factors that are associated with a poor outcome following CRS include *(i)* presentation with an obstructed or perforated primary tumor, *(ii)* high PCI, and *(iii)* completeness of CRS. The currently used in- and exclusion criteria for surgical treatment are solely based on PCI, judgement of resectability, and the presence of distant metastases outside the peritoneum. Despite these selection criteria, approximately half of the patients experience rapid disease progression within the first year after CRS-HIPEC, which further increases to 70% after 2 years (50). Eventually, recurrence after CRS-HIPEC results in 2- and 10-year overall survival rates of ~60% and ~20% respectively (50–53).

The currently used selection criteria carry no biological information, including for instance, genetic or histological subtypes, or intra-abdominal location. Such variables may have a profound impact on tumor behavior and recurrence rates. A major challenge therefore is to identify tumor- or patient-centric biological and/or genetic variables that are associated with recurrence following CRS-HIPEC (Figure 1).

Accurate prediction of benefit from CRS will require the design of composite biomarkers in which a robust (imaging-based) PCI scoring system is combined with the relevant clinical and biological/genetic parameters. Such biomarkers may then be used to create decision tools balancing clinical benefit and morbidity in order to improve the selection of patients for surgical treatment.

### Optimizing CRS: Detection of Micro-Metastases

The success of surgical treatment is mostly determined by the completeness of resection. A more effective approach to radically remove intraperitoneal micro-metastases would be to identify them using intra-operative imaging, which has the potential to guide surgery (54–57). Clinical feasibility of that process has now been demonstrated (56, 57). Encouragingly, systemic administration of a fluorescent anti-CEA monoclonal antibody was safe and allowed subsequent intra-operative detection of PM. Importantly, this technique not only identified superficially growing metastases, but also more deeply situated metastases that would otherwise have remained undetected, resulting in a change of the surgical strategy in about one third of the cases (56). This study demonstrates proof-of-concept that intra-operative imaging has the potential to improve the completeness of resection. Harnessing this technology for improving clinical outcome will require a further identification of relevant targets expressed on PM from CRC, and optimization of tracer backbones, imaging modalities, and routes of administration (Figures 1, 2). An important question is whether microscopic intraperitoneal target lesions have sufficient functional blood vessels to allow their detection by systemically administered tracers.

### Optimizing the HIPEC Procedure

The combination of CRS and HIPEC in patients with PM from CRC can result in 5-year survival rates of over 40% (50, 51, 53). A major question is whether this is due to CRS alone, or whether HIPEC has additional value. A clear benefit of the HIPEC procedure following CRS has recently been demonstrated in patients with PM from ovarian cancer (58). However, the PRODIGE7 trial failed to prove an additional value of HIPEC in patients treated for PM from CRC (47). However a *post-hoc* subgroups analysis showed that HIPEC improved relapse-free and overall survival specifically in a subgroup with intermediate PCI (11–15), suggesting that future optimization of the procedure should focus on this subgroup with the potential for (near-) complete surgical PM resection. In general, one has to realize that the PRODIGE7 trial was performed using a high-dosed, short duration (30 min) oxaliplatin-based HIPEC-regimen in patients that were selected after 6 months of systemic treatment with oxaliplatin-containing regimens. The additional value of other HIPEC-regimens (including those with rationally selected drug combinations) in colorectal cancer patients has not been addressed in randomized trials yet.

Pre-clinical studies with PM-derived organoids have indicated that heated Oxalipatin or MMC monotherapies do not achieve their eradication (38). Despite their highly heterogeneous presentation, PM from CRC are mostly of the CMS4 molecular subtype (20). Importantly, CMS4 CRC do not benefit from the addition of oxaliplatin to adjuvant therapy (59, 60). Thus, while the concept of HIPEC after CRS appears to be valid and can result in a significant survival benefit in ovarian cancer (58), the currently used drugs in HIPEC for PM from CRC are unlikely to be effective as monotherapies.

Optimization of the HIPEC regimen is therefore a major challenge (Figure 1). Several approaches could be considered. First, drug combinations that are capable of killing CMS4-type micro-metastases in a very short period of time (<90 min) need to be identified. In addition, the added value of heat should be assessed and, in the case of mucinous tumors, a mucus (pre-) clearing approach could be considered. The feasibility and safety of intra-abdominal mucin dissolution by combined administration of bromelain and acetylcysteine in cancer patients with mucinous PM was recently demonstrated (61). Finally, alternative drug formulations that prolong intra-peritoneal drug exposure times (e.g., using nanoparticles, hydrogels or albumin) forms an attractive innovative approach to improve local drug efficacy (62, 63).

### Peri-Operative Chemotherapy

While systemic chemotherapy has prolonged the survival of patients with inoperable PM, this benefit is markedly higher for patients with non-PM distant metastases (2, 9). The added benefit of standard systemic peri-operative chemotherapy over CRS-HIPEC alone is currently being investigated in a large phase 3 trial using MMC-based HIPEC (CAIRO6) (64).

A third approach to target microscopic tumor residue is to improve (neo-) adjuvant treatment regimens with chemo- and/or targeted therapy. Key questions relating to this approach are *(i)* Do intra-peritoneal micro-metastases have sufficient functional blood vessels to allow systemically administered drugs to reach them and sort a beneficial effect?, and *(ii)* Does the intra-peritoneal location of PM generate specific therapeutically exploitable vulnerabilities?

### Systemic Chemotherapy for Inoperable Disease

Systemic therapy is widely used to treat metastatic CRC. The most effective regimens have prolonged median overall survival to more than 2 years (65). However, the benefit of systemic therapy (including regimens with novel targeted agents) is dramatically reduced in patients with PM involvement, both in terms of response and survival (2). It is currently unknown which factor(s) cause the site-dependent differential response to systemic therapy. Such factors may include site-specific differences in drug delivery and tumor penetration, tumor biology, intrinsic resistance [e.g., caused by CMS4 status (20) or mutant BRAF (1)], and drug metabolism. Insight into the factors that cause the relative resistance of PM to current systemic therapies is central to the design of more effective systemic treatment regimens (Figures 1,2).

### Intraperitoneal Chemotherapy

The enclosed nature of the peritoneal space offers the unique but underexplored possibility to design and test novel intra-peritoneal treatment strategies. The limited systemic uptake of intraperitoneally delivered drugs further allows the use of much higher drug concentrations than could ever be administered systemically. Furthermore, PM are directly exposed to the drugs, which could help target even poorly vascularized tumors.

Pressurized intraperitoneal aerosolized chemotherapy (PIPAC) is used to achieve a homogenous spread of chemotherapy throughout the peritoneal cavity. The procedure is safe and allows repeated cycles of treatment (66). Thus far, PIPAC has been based on oxaliplatin monotherapy. However, oxaliplatin monotherapy has never been demonstrated to be effective in the systemic treatment of distant metastases. Moreover, as outlined above, both experimental and clinical evidence indicate that it is unlikely that oxaliplatin monotherapy will be an effective treatment against PM from CRC given the intrinsic resistance that is associated with their CMS4 status (38). In order to fully exploit the potential benefits of the procedure more effective drug combinations need to be identified and tested. Small proof-of-concept PIPAC studies involving tens of patients are ideally suited to test such novel treatment combinations–e.g., identified on the PM organoid platform–in a relatively short period of time.

An alternative procedure involves repeated infusion of drugs into the abdominal cavity via a peritoneal access port. In the INTERACT study this approach is investigated using irinotecan as monotherapy (67). Irinotecan monotherapy has demonstrated value in the systemic treatment of distant metastases (68). However, differences in the mode of administration, the timing of treatment, and biological differences between PM and metastases at other sites may all have an impact on how metastases respond to irinotecan monotherapy.

The PIPAC and INTERACT designs and the enclosed nature of the peritoneal cavity offer the exciting possibility to rapidly test existing and novel drugs, alone and in combination, for their anti-PM activity in relatively small patient groups. Realistically, it must be possible to identify local PM-targeting regimens that are more effective than the currently used monotherapies (next section, Figure 2).

## Future Directions

### Understanding the Biology of PM

The evolutionary history of metastases is a topic of intense research in many types of cancer (29, 69, 70). Models of tumor evolution describe the processes that lead to the generation of metastasis-competent clones in primary tumors, the timing of their dissemination, and the factors determining successful outgrowth at distant sites. Primary tumors that have breached the colon wall have immediate access to the peritoneal cavity. Therefore, PM formation may not require intra- and extravasation steps. Furthermore, the PM-seeding entities are clusters of tumor cells that bud off from the primary tumor (71). Cancer-associated fibroblasts within the peritoneal micro-environment may play an important role in the process of peritoneal seeding of cancer cells (72). For a detailed description of the role of the microenvironment in PM formation, we refer the reader to an excellent recent review on this topic (73). Apparently, a single cell stage is also not required during this mode of dissemination. Interestingly, tumors that have not completely breached the colon wall (i.e., T1-T3) frequently also give rise to PM (74). This may be due to sampling bias that is intrinsic to histology-based TNM staging. Alternatively, cancer cells may exfoliate from the primary tumor during surgery and subsequently seed the peritoneum. Nevertheless, it is also conceivable that alternative biological routes to PM formation exist that are currently unknown. Determination of the evolutionary relationships between multiple primary tumor regions, PM and metastases at other sites will provide insight into this matter.

Pseudomyxoma Peritonei (PMP) represents a unique form of PM with unique clinical challenges and underlying biology. We refer the reader to a focused recent review on PMP discussing the biology underlying PM formation and the consequences for treatment (75).

The micro-environments in distant organs are highly distinct. Therefore, the phenotypic and genotypic traits that are selected for during metastasis formation are likely to be site-dependent. However, whole genome sequencing has demonstrated that the driver mutations in distant non-PM metastases are highly homogeneous within individual patients, presumably because they were all derived from a specific sub-clone within the primary tumor (76–78). Despite these overall similarities, site-specific genetic differences affecting distinct biological processes exist between CRC liver and brain metastases, illustrating the principle of site-specific adaptation (79, 80). Very little is known about the evolutionary processes (mutations, copy number alterations, epigenetic modifications, etc.) that specifically shape the PM-competent tumor genome. Nevertheless, specific histological subtypes–in particular mucinous adenocarcinoma and signet ring cell carcinoma–show a remarkable preference for metastasizing to the peritoneum. In addition, tumors with activating mutations in *BRAF*, and those of the CMS4 molecular subtype are also prone to form PM. Understanding why these specific histological and genetic tumor subtypes are associated with PM formation will provide leads to the design of novel therapies and diagnostic tools.

Even within the peritoneum the requirements for successful site-specific adaptation may be different depending on the site within the peritoneum, such as omental fat, diaphragm, and the surface of intra-abdominal organs. Processes governing site-specific adaptation (through whatever evolutionary mechanism) and (epi)genetic diversity within and between PM are likely to be highly relevant for the potential success of intraperitoneal therapies. Metabolic adaptation of PM to the fatty acid-rich microenvironment of the abdomen may create a targetable, generic, PM-specific vulnerability (81).

Finally, while the vast majority of studies on metastasis formation focus on tumor-centric parameters, the process is also likely to be influenced by genetic and epigenetic variation in the human population (29, 82). Therefore, a full understanding of the biology of PM will require (epi-)genetic analyses of both the tumor and the patient.

### A Research Framework to Meet the Key Challenges

An in-depth insight into the biology of PM will greatly help in meeting the clinical challenges listed in Figure 1. We envision a research framework that is built on the acquisition of tumor tissue, ascites, blood, and normal tissues of patients with PM from CRC (Figure 2). Whenever and wherever possible tissues and ascites should be collected in a longitudinal fashion, allowing the in-patient analysis of tumor progression and response to treatment over time. Direct cross-omics analysis of multiple lesions and paired primary tumor samples (derived, for instance, from CRS procedures) will give unprecedented insight into many aspects of PM biology, including their evolutionary history, the genetic and epigenetic intra- and inter-tumor heterogeneity, the association with specific (epi-) genetic features, expression of PM-specific (cell surface) markers, activation of specific signaling and metabolic pathways, molecular subtypes, etc. These data will form a solid basis for designing effective PM-specific detection and treatment strategies. For example, the finding that PM are largely of the CMS4 molecular subtype and frequently carry activating mutations in the BRAF oncogene have already provided two new leads for developing and testing alternative therapeutic approaches. For instance, TGFb is a central player in aggressive CRC (83) and orchestrates tumor-associated fibrosis and fibroblast-cancer cell crosstalk, which is a hallmark of CMS4 CRC (83–85). Multiple TGFb-targeting agents are under development for the treatment of CRC (trial numbers: NCT03470350; NCT03436563) and may be especially effective in targeting CMS4 CRC, including PM.

Pre-clinical evaluation of the potential value of any new treatment strategy requires the availability of clinically relevant model systems. The pre-clinical model systems that have been used to date are based on traditional CRC cell lines *in vitro* or injected into the peritoneal cavity of mice or rats. Unfortunately, this approach does not take into account that PM are a highly specific disease entity with features that are not necessarily recapitulated in traditional cell line models. Moreover, the culturing technique that is used to establish traditional cell lines causes profound genomic rearrangements, resulting in the outgrowth of highly selected sub-clones poorly resembling the original tumors. Therefore, the validity of these models in studying PM biology is questionable.

As an alternative, we propose that the tissues and ascites samples that are derived from patients with PM can be used to create representative PM model systems, particularly based on organoid technology (37, 38). Organoid technology is now widely considered to be the most relevant culturing method for many types of cancer and normal tissues as it preserves the genetic and phenotypic characteristics of the original (tumor) tissue and allows limitless expansion of the tissue in culture (39). Organoid cultures capture (at least to some extent) the functional and genetic heterogeneity that is characteristic of CRC (86, 87). Furthermore, organoids can also be used to generate spontaneous metastasis models by making use of microsurgical transplantation techniques (88). Organoid technology has already proven to be of clinical relevance in primary and metastatic CRC as the treatment responses observed in organoids closely resemble those observed in cancer patients, in multiple independent studies (40–43). However, PM have so far not been included in these studies. We and others have recently shown that organoid technology can be used to create novel PM models and that these models have the potential to improve the treatment of PM either through a rational approach of drug selection and testing or, via unbiased drug screens (37, 38). Indeed, drug screens on PM-derived organoids have the potential to identify alternative personalized treatment options for patients in whom standard of care treatment failed (37).

Ideally, all histological, molecular and genetic subtypes are represented in a PM organoid biobank. Organoid generation from all PM subtypes and from all-intra-peritoneal locations may require further optimization of culturing conditions. The results obtained from direct analysis of the PM tumor tissue samples may inform such adaptation of culture conditions. For instance, specific niche factors and/or stromal cell populations (e.g., adipocytes, fibroblasts, mesothelial cells, factors present in ascites, etc.) may be tested for their influence on organoid growth and behavior and response to therapy. Ultimately, the value of PM-derived organoid models in predicting therapy response should be tested in clinical trials, similar to those performed in patients with non-PM primary and metastatic CRC (40–43). Clearly, this will also require optimization of the detection of intraperitoneal drug responses. DW-MRI, or molecular imaging based on expression of FAP, may have sufficient specificity and selectivity to design such trials (14, 15). In addition, PM-specific markers or metabolic pathways may be identified based on PM tissue analysis. These could serve as targets for developing alternative methods to detect and treat PM.

Organoid-based models (*in vitro* and in animals) are also ideally suited to study novel drug combinations, guided by the results from direct PM analysis (see above). The efficacy of any drug on any cell type is directly correlated to the time of exposure. Drug exposure times during the currently used HIPEC procedures is very short (30–90 min). Novel nanoparticle-, hydrogel- or albumin-based drug formulations may be used to improve local treatment efficacy simply by prolonging the time of drug exposure following a similarly short procedure (62). In particular, hydrogels with self-healing properties hold great promise in this area and should be tested in *in vitro* and *in vivo* pre-clinical models using PM-derived organoids.

Another issue that needs resolving is the added value of heat. From the first clinical trials exploring the benefit of HIPEC, heat has always been an integral part of the treatment strategy (51, 52, 89) but the added value of heat over drug treatment alone has never been assessed. Of note, heat may compromise drug activity and this should be evaluated for any candidate drug to be included in the HIPEC procedure. The PM-specific model systems can now be used to directly address this question without having to design clinical studies. The natural cellular defense response against heat involves activation of the ATR DNA damage checkpoint pathway (90), for which many drug are available. Indeed, ATR inhibition showed great synergy in killing PM-derived organoids in combination with the standard-of-care drug MMC at 42°C (38). To maximize the anti-tumor effects of heat application during HIPEC this concept–and others–must now be further tested on the organoid platform, and in pre-clinical animal models with organoid-initiated PM.

## Conclusions

We foresee that the recent developments in the field that are described here, in particular the application of organoid technology, the use of DW-MRI and molecular imaging for PM detection, the further improvement of intra-peritoneal drug delivery, and the design of novel clinical proof-of-concept studies, will drive innovation in the clinical management of PM from CRC. An extensive integration of research and care will continue to be required to gain in-depth insight into the biology of PM, and this will form the basis for designing and testing better PM detection and treatment strategies. We hope that the research framework described here will help structure the efforts toward reaching that goal.

## Author Contributions

All authors contributed to the conceptualization and writing of the manuscript.

## Conflict of Interest

The authors declare that the research was conducted in the absence of any commercial or financial relationships that could be construed as a potential conflict of interest.
